# Comparison of Poisson and Bernoulli spatial cluster analyses of pediatric injuries in a fire district

**DOI:** 10.1186/1476-072X-7-51

**Published:** 2008-09-22

**Authors:** Craig R Warden

**Affiliations:** 1 Departments of Emergency Medicine & Pediatrics, Oregon Health & Science University, Portland, Oregon, USA; 2 Department of Geography, Portland State University, Portland, Oregon, USA

## Abstract

**Background:**

With limited resources available, injury prevention efforts need to be targeted both geographically and to specific populations. As part of a pediatric injury prevention project, data was obtained on all pediatric medical and injury incidents in a fire district to evaluate geographical clustering of pediatric injuries. This will be the first step in attempting to prevent these injuries with specific interventions depending on locations and mechanisms.

**Results:**

There were a total of 4803 incidents involving patients less than 15 years of age that the fire district responded to during 2001–2005 of which 1997 were categorized as injuries and 2806 as medical calls. The two cohorts (injured versus medical) differed in age distribution (7.7 ± 4.4 years versus 5.4 ± 4.8 years, p < 0.001) and location type of incident (school or church 12% versus 15%, multifamily residence 22% versus 13%, single family residence 51% versus 28%, sport, park or recreational facility 3% versus 8%, public building 8% versus 7%, and street or road 3% versus 30%, respectively, p < 0.001). Using the medical incident locations as controls, there was no significant clustering for environmental or assault injuries using the Bernoulli method while there were four significant clusters for all injury mechanisms combined, 13 clusters for motor vehicle collisions, one for falls, and two for pedestrian or bicycle injuries. Using the Poisson cluster method on incidence rates by census tract identified four clusters for all injuries, three for motor vehicle collisions, four for fall injuries, and one each for environmental and assault injuries. The two detection methods shared a minority of overlapping geographical clusters.

**Conclusion:**

Significant clustering occurs overall for all injury mechanisms combined and for each mechanism depending on the cluster detection method used. There was some overlap in geographic clusters identified by both methods. The Bernoulli method allows more focused cluster mapping and evaluation since it directly uses location data. Once clusters are found, interventions can be targeted to specific geographic locations, location types, ages of victims, and mechanisms of injury.

## Background

Analysis using geographical information systems (GIS) is just beginning to be tapped in the field of injury prevention.[1] Injuries are most likely spatially heterogenous with some mechanisms constrained geographically, for example, motor vehicle collisions and bicycle and pedestrian injuries will only occur on a roadway. Prevention strategies need to be targeted as much as possible due to constraints on resources available. Fire district resources are assigned to permanent stations and response areas and are limited in the distance they can travel for non-emergency tasks such as injury prevention talks and inspections. If a crew is to be assigned to injury prevention interventions, they need geographic information on where injuries are occurring and where potential target populations are accessible. Even funding of an independent entity such as an academic injury prevention program is limited and its efforts need to be targeted to specific geographic areas and populations to be cost effective.

One study of fall-related injuries in central Toronto used GIS to demonstrate that in addition to age and household income census tract data, the location of homeless shelters appeared to be significantly associated with the distribution of injuries.[2] Motor vehicle collisions cause more deaths in children < 15 years old than any other cause and have diverse geographic variation across the US.[3] A Canadian national study of adolescent injuries revealed a disparity in injury rates from urban (lower rate) to rural (higher rate) populations.[4] Fall-related injuries are the most frequent mechanism of pediatric injuries in the US, though with lower mortality rates than motor vehicle collisions.[5] These injuries can still cause significant head injuries that can affect future cognitive function. A study of pedestrian-related injuries in Montréal using ambulance service data showed that only 1% of intersections had at least one victim and these accounted for only 4% of all injured pedestrians.[6] This is illustrative of the difficulty in attempting to target a limited number of intersections for pedestrian injury prevention. These studies show that there is a clear spatial component to injury patterns and different mechanisms of injury need to be accounted for in the analysis.

The spatial statistic SaTScan™ using the Poisson method has had wide acceptance in detecting disease clusters in many different situations. [7-11] It has been found to have reasonable sensitivity and specificity when compared to generalized additive models (GAM) and Bayesian disease mapping[12] and to the Besag-Newell's R, Cuzick-Edwards' k-Nearest Neighbors, Tango's Maximized Excess Events Test, and Moran's I [13] in cluster models. The Bernoulli method (see Methods section) in SaTScan has been used to identify census tracts with clusters of high metastatic versus localized prostate cancer incidence in the state of New Jersey with success.[14] To my knowledge, it has not been used for modelling traumatic injury geographical patterns.

The objective of this study is to evaluate and compare the Poisson and Bernoulli methods in SaTScan in finding potential geographical clusters of pediatric injuries within a fire district's boundary. This fire district is very active in injury prevention activities and wishes to see if it can focus its interventions more effectively using these methods. If significant clusters are found, the next step is to evaluate potential injury prevention strategies depending on the characteristics of injuries in each cluster.

## Methods

### Study area

**Figure 1:**
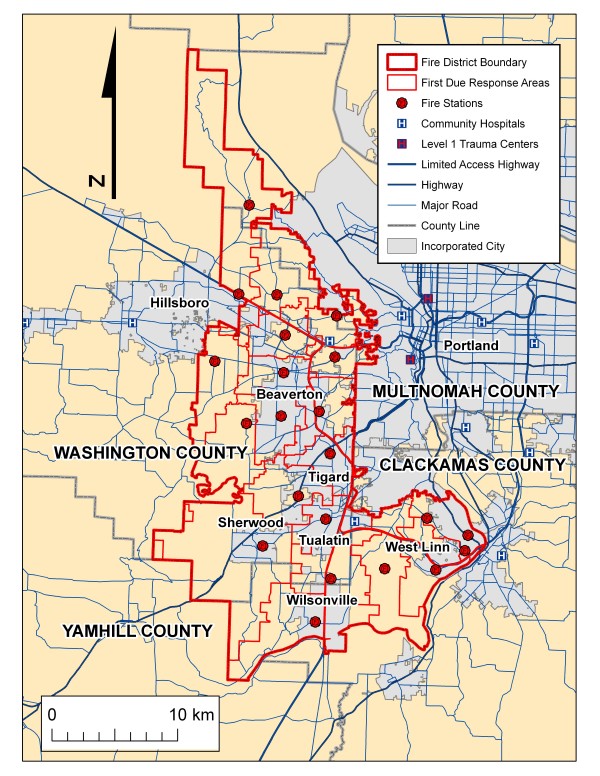
**Study area: Tualatin Valley Fire & Rescue District (TVF&R)**. This map shows the boundary of the fire district, the major cities, county boundaries, fire station locations and their respective first-due response areas, the major highway and street system, and the location of community hospitals and the two pediatric trauma centers.

The Tualatin Valley Fire and Rescue (TVF&R) district consists of an oblong shaped area of 210 square miles (544 square kilometres) serving a population of approximately 369,000 based on 2000 census data with 22 fire stations and 28 first-line fire apparatus (Figure 1). The district is located to the west of the city of Portland, Oregon serving a large portion of Washington County and portions of Clackamas County and includes a mix of urban, suburban and rural areas. It responds to approximately 31, 000 emergency medical services (EMS) incidents a year. The fire district has a robust data collection process including the automatic geocoding of all emergency response locations creating a rich database for GIS analysis. TVF&R responds to all 9-1-1 generated emergency responses within its boundaries.

### Study population

All patients less than 15 years of age that had an emergency medical response within the current boundaries of the TVF&R district during 2001–2005 were included.

### Data collection

Patient data including location of call is documented in an electronic charting system (Sunpro,™ Aether Systems, Inc., Baltimore MD) by treating firefighters. Patients' home addresses are not routinely recorded if the incident did not occur there. This database was then queried and the data downloaded as an Excel 2003™ (Microsoft, Inc., Redmond WA) spreadsheet. Variables analyzed were patient demographics, location of incident, location type, and mechanism of injury. No patient identifying data was available to the author. The Oregon Health & Science University Institutional Review Board approved this study.

### Data analysis

#### Base map and patient data analysis

The mechanisms of injuries were aggregated into motor vehicle collision injuries (patients that were passengers in the motor vehicle); bicycle and pedestrian injuries (motor vehicle versus bicyclist or pedestrian or a fall off a bicycle); fall injuries; assault injuries (intentional including alleged child abuse); and "environmental" (poisoning, heat or cold injuries, drowning and burns) to aggregate similar mechanisms to ensure adequate cases in each cohort for analysis. Separate shapefiles were created for the total injuries cohort and each of the mechanisms above. Location type information was consolidated into 1) school, church, or day care; 2) multifamily residential building; 3) single family residence; 4) sport or recreational facility or park;[15,16] 5) other public building; or 6) street or road. Age was also analyzed by stratifying into age groups defined by the Centers for Disease Control.[17,18] Disposition was defined as died at scene, not transported, and transported to a hospital (the only options in the EMS system). Race or ethnicity was classified as white, Hispanic, African-American and other or missing together. Patient data was imported into SPSS™ 15.0 (SPSS, Inc., Chicago IL) for statistical analysis. The characteristics of the medical and injured cohorts were compared using the t-test or Pearson χ^2 ^statistic where appropriate.

Base map data layers included the fire district's boundary, station locations and first-due areas, census tract boundaries and population, city and county boundaries (Portland Metro Data Resource Center), and streets and highways (StreetMaps,™ ESRI, Inc., Redlands WA). All maps and analysis used the NAD 1983 HARN State Plane of Oregon North FIPS 3601 coordinate system (Lambert conformal conic projection).

The fire district automatically geocodes incident locations as part of their data management using ArcInfo™ (Environmental Systems Research Information, ESRI, Inc., Redlands CA). The fire district GIS analyst manually locates unmatched incidents from the automatic geocoding and these were not validated independently by the author. The patient location data was transferred as a point shapefile and matched to patient case files using a unique patient incident number. The author imported this patient location and case data into ArcView™ 9.2 (ESRI, Inc., Redlands WA) for geographical analysis.

#### Bernoulli cluster analysis method

For both methods of cluster detection, SaTScan uses a moving, varying diameter window to evaluate clusters. For each window location and size, the software calculates the number of observed and expected observations inside the window and, in turn, calculates the likelihood function for each window, the form of which differs depending on the assumed distribution of events. For the Bernoulli model, the two patient cohorts (medical and injury) were split into separate point shapefiles for comparison. The two patient cohorts were then imported into SaTScan™ (National Cancer Institute, Division of Cancer Control and Population Sciences, Statistical Research and Applications Branch) and analyzed using the Bernoulli method.[8,14,19,20] Briefly, the Bernoulli model uses two cohorts of cases and controls to determine if there is significant clustering of the case location distribution as compared to the controls location distribution.[7,10] The advantage of this method is that it is independent of the underlying population distribution. In disease processes such as injuries the potential causes may not be distributed similarly to the population at risk ie. motor vehicle collisions and pedestrian injuries occur along road networks that may not reflect the underlying population at risk. In addition, population density distributions can be only be approximated from census data especially in rural or industrial areas. The SaTScan Bernoulli model uses a likelihood ratio test of the probability of a group of patients within a potential cluster defined by a circle being a case versus a control. The likelihood function for the Bernoulli model is:

$${\left(\frac{c}{n}\right)}^{c}{\left(\frac{n-c}{n}\right)}^{n-c}{\left(\frac{C-c}{N-n}\right)}^{C-c}{\left(\frac{\left(N-n\right)-\left(C-c\right)}{N-n}\right)}^{\left(N-n\right)-\left(C-c\right)}$$

where C is the total number of cases, c is the observed number of cases within the window, n is the total number of cases and controls within the window, N is the combined total of cases and controls within the data set, and I () is the indicator function which is equal to 1 if c > C/N or 0 otherwise. Since this analysis is only interested in detecting clusters with higher than expected rates, I () was set equal to 1.

#### Poisson cluster analysis method

**Figure 2:**
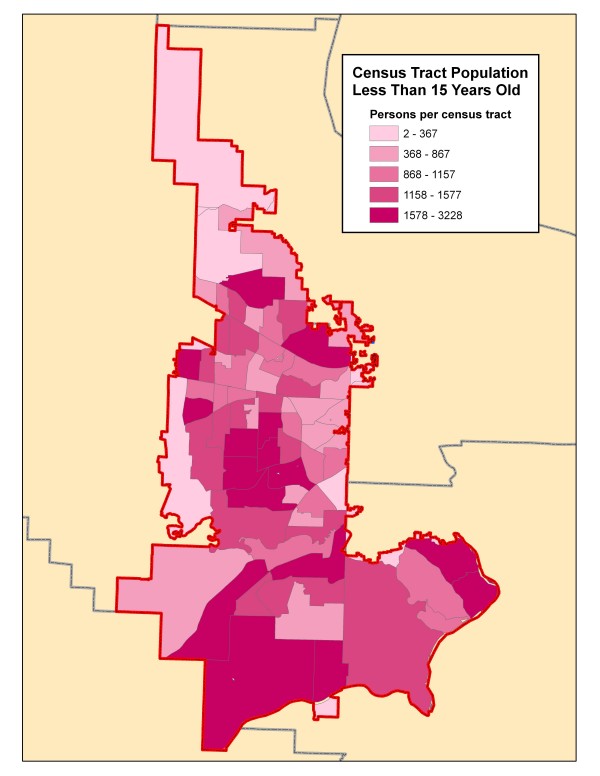
**Census tract population less than 15 years old**. This map demonstrates the population of each census tract or partial census tract of children less than 15 years old for the year 2000. The tracts are shaded according to the quantile method.

For the Poisson method in SaTScan, population data at the census tract level was used. Exploratory data analysis showed this to be the lowest level of aggregation that would generate reasonable incidence rates for analysis. Population data for the 2000 Census was downloaded from the Census Bureau  and joined to the census tract polygon shapefile. The population of children less than 15 years old was calculated for each census tract by aggregating all the appropriate age groups. Since the fire district boundary does not align with the census tracts, it was overlaid on the census tract polygon layer and four resultant "sliver" census tract polygons of less than one hectare and with no incidents were eliminated from this part of the analysis (Figure 2). For the remaining partial census tracts, the pediatric population at-risk was estimated using the remaining area and assuming a uniform population density. The incidence of injury incidents per census tract was calculated using the "point-in-polygon" method of overlaying the respective incident locations and census tract polygon and its population at-risk. This was done for the total injuries cohort and each mechanism cohort for separate cluster analyses. For the SaTScan Poisson analysis, centroids of the census tracts were used to define the location of the population at-risk and injury cases that occurred within the census tract.

Under the null hypothesis for the Poisson model, the expected number of cases in each census tract is proportional to the population size. For this analysis temporal data was not taken into account. Under the Poisson assumption, the likelihood function for a specific window is proportional to:

$${\left(\frac{c}{E[c]}\right)}^{c}{\left(\frac{C-c}{C-E[c]}\right)}^{C-c}I()$$

where C is the total number of cases, c is the observed number of cases within the window, E [c] is the expected number of cases within the window under the null hypothesis, and I () is the indicator function which is equal to 1 if c > E [c] or 0 otherwise. Since this study is only interested in detecting clusters with high rates, I () was set equal to 1. [7]

For both the Poisson and Bernoulli models, the likelihood ratio is tested for significance using the Monte Carlo method. A circular window is centered on each census tract centroid (for Poisson analysis) or each incident location (for Bernoulli analysis) and the diameter is varied from zero to one that includes *a priori *a certain maximum proportion of the total number of case events. For the purposes of this analysis, 999 Monte Carlo replications were used, the maximum circle size included up to 50% of the total cases being analyzed, and a significant p-value was less than 0.05. The likelihood function is maximized over all window locations and sizes and the one with the maximum likelihood constitutes the most likely cluster. Secondary non-overlapping clusters can then be found by subtracting the most likely cluster cases (and controls in the Bernoulli method) from the pool and repeating the above procedure. Any edge effect was ignored in this analysis since there was no data available from outside the fire district's boundary.

## Results

### Descriptive

There were an estimated 82, 400 children less than 15 years old living within the fire district boundary. During the study period, the fire district responded to a total of 2806 medical calls and 1997 injuries in patients less than 15 years of age for an incidence of 6.8/1000/year and 4.8/1000/year, respectively. Figure 2 demonstrates the census tract population distribution for children less than 15 years old in the fire district. The intervals for the choropleth map were determined by the quantile method. Aggregating similar injury mechanisms revealed there were 413 injuries due to motor vehicle collisions, 219 due to pedestrian and bicycle injuries, 1035 due to falls, 236 due to environmental injuries, and 94 due to assaults.

### Bernoulli cluster analysis

**Figure 3:**
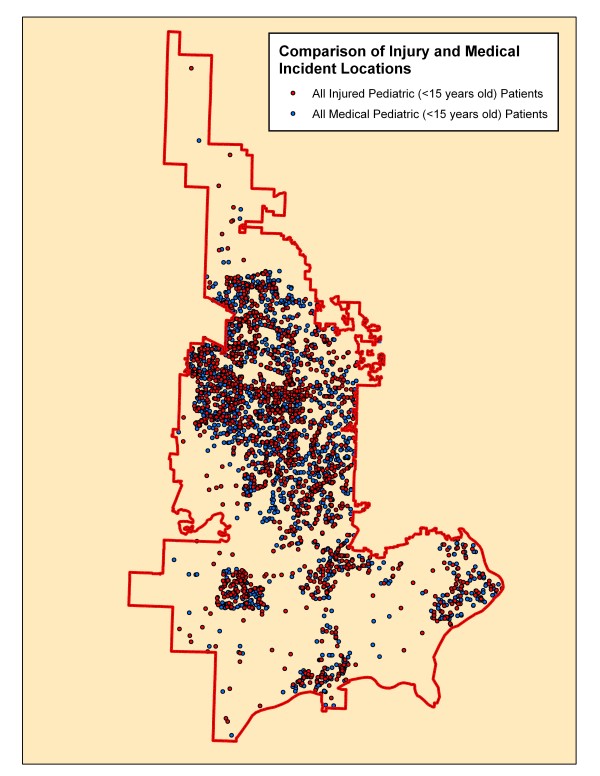
**Distribution of all injuries and medical calls locations**. This map reveals a subtle difference in the distribution of these two cohorts but both are primarily located in population centers and along the road and street network when superimposed on these.

Table 1 compares the demographics, incident location and disposition of the injured versus medical cohorts of patients. Not surprisingly, the injured patients were older and were found in more public locations and roadways. As children age, they are more prone to injuries due to being more independent and being transported by motor vehicles more.[21] Medical illness related calls would not be expected to occur *on *the road and street network. There was a slight difference in the racial and ethnic distribution but with the significant amount of missing data making it difficult to interpret. Fewer injured patients were transported by EMS than for medical reasons. This may be due to calls being initiated by bystanders for relatively minor injuries but this is conjecture at this point. Figure 3 demonstrates the distribution of the location of injury related and medical related incidents.

**Table 1:** Comparison of medical and injured patient cohorts

	**Medical Patients # 2806**	**Injured Patients # 1997**	P-value*
***Demographics***			
**Age**	5.4 ± 4.8 yrs	7.7 ± 4.4 yrs	< 0.001 (t-test)
**Age Groups**			
**0 – 12 mos**	23%	8%	
**1 – 4 yrs**	32%	24%	< 0.001
**5 – 9 yrs**	23%	36%	
**10 – 14 yrs**	23%	36%	
**Male**	57%	56%	0.62
**Race**			
**Other/Missing**	23%	19%	
**African- American**	4%	4%	0.001
**Hispanic**	11%	11%	
**White**	62%	66%	
***Location Type***			
**School/Church/Daycare**	12%	15%	
**Multi-family building**	22%	13%	
**Single family residence**	51%	28%	< 0.001
**Sport/Park/Recreation**	3%	8%	
**Public building**	8%	7%	
**Street/Road**	3%	30%	
***Disposition***			
**Died**	0.5%	0.3%	
**No transport**	35%	44%	< 0.001
**Transported**	64%	56%	

*(χ^2 ^test except as noted)

**Figure 4:**
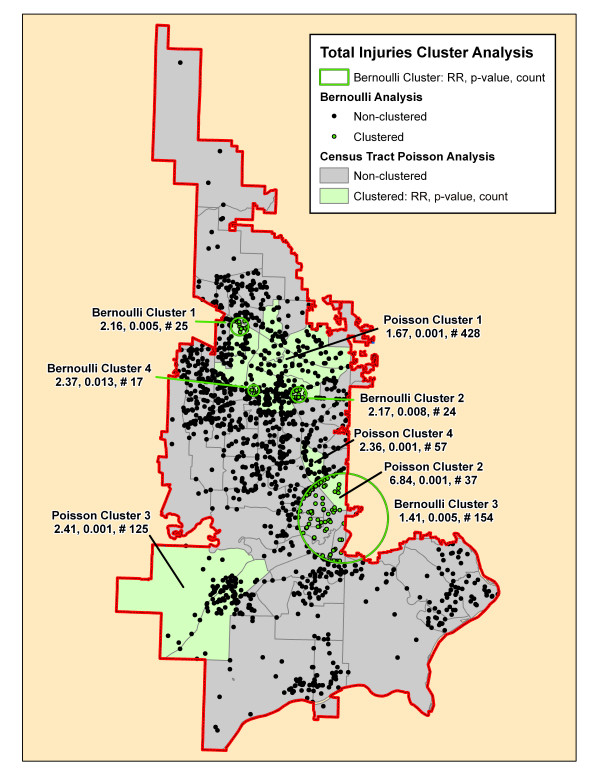
**Total injuries cluster analysis**. The significant clusters by the SaTScan Poisson (green-shaded census tracts) and Bernoulli (green circles and green incident locations) methods are superimposed on this map. Each cluster is identified by its rank followed by its relative risk (RR), associated p-value and the number of cases in the cluster.

**Figure 5:**
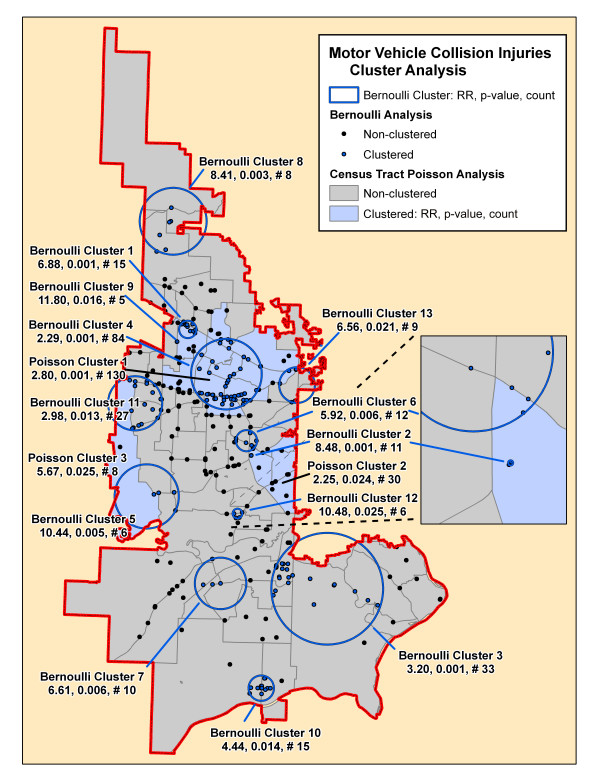
**Motor vehicle collision injuries cluster analysis**. The significant clusters by the SaTScan Poisson (blue-shaded census tracts) and Bernoulli (blue circles and blue incident locations) methods are superimposed on this map. Each cluster is identified by its rank followed by its relative risk (RR), associated p-value and the number of cases in the cluster.

**Figure 6:**
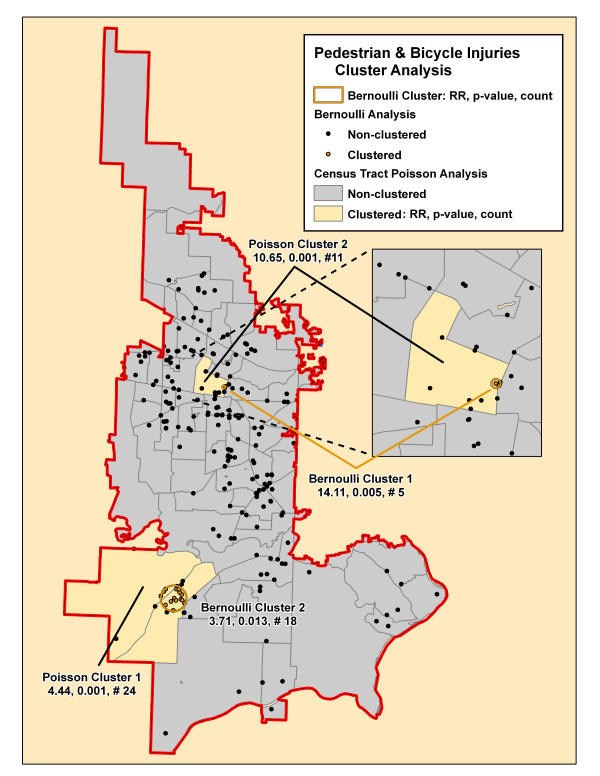
**Pedestrian and bicycle injuries cluster analysis**. The significant clusters by the SaTScan Poisson (yellow-shaded census tracts) and Bernoulli (orange circles and orange incident locations) methods are superimposed on this map. Each cluster is identified by its rank followed by its relative risk (RR), associated p-value and the number of cases in the cluster.

**Figure 7:**
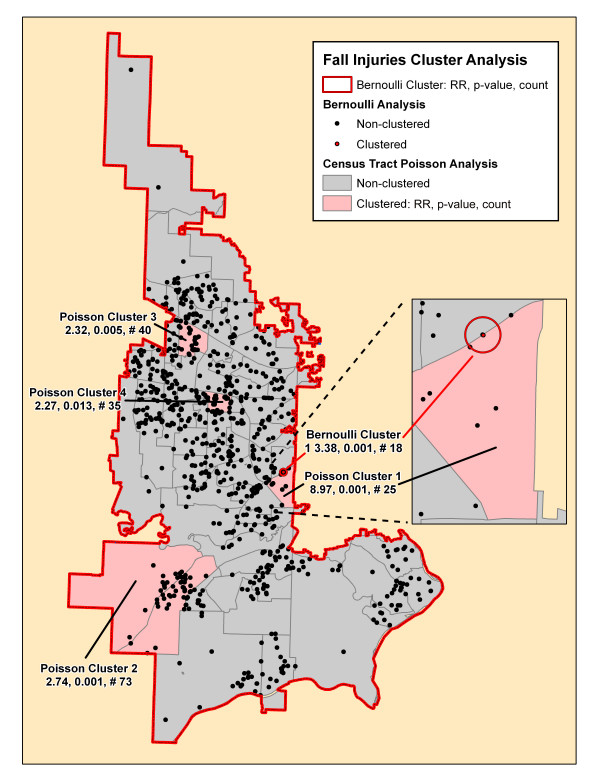
**Fall injuries cluster analysis**. The significant clusters by the SaTScan Poisson (pink-shaded census tracts) and Bernoulli (red circles and red incident locations) methods are superimposed on this map. Each cluster is identified by its rank followed by its relative risk (RR), associated p-value and the number of cases in the cluster.

**Figure 8:**
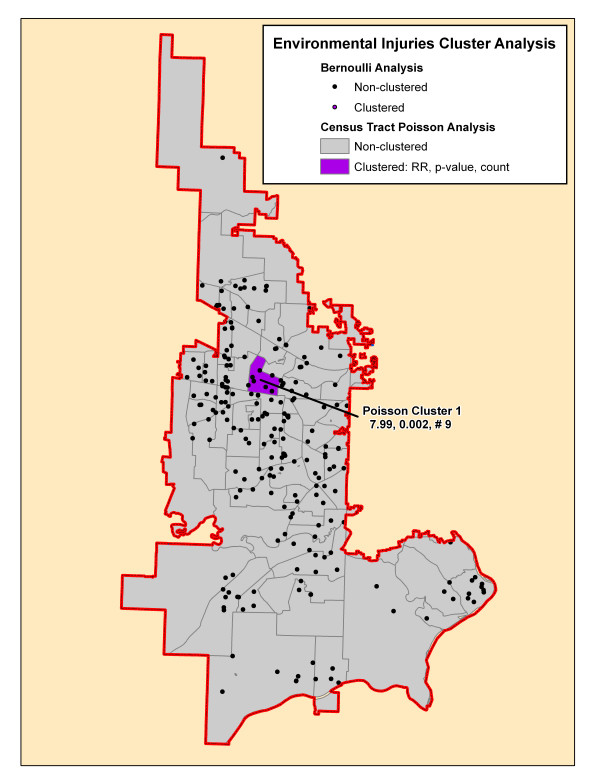
**Environmental injuries cluster analysis**. The significant cluster by the SaTScan Poisson (purple-shaded census tract) on this map is presented on this map. The cluster is identified by its rank followed by its relative risk (RR), associated p-value and the number of cases in the cluster.

**Figure 9:**
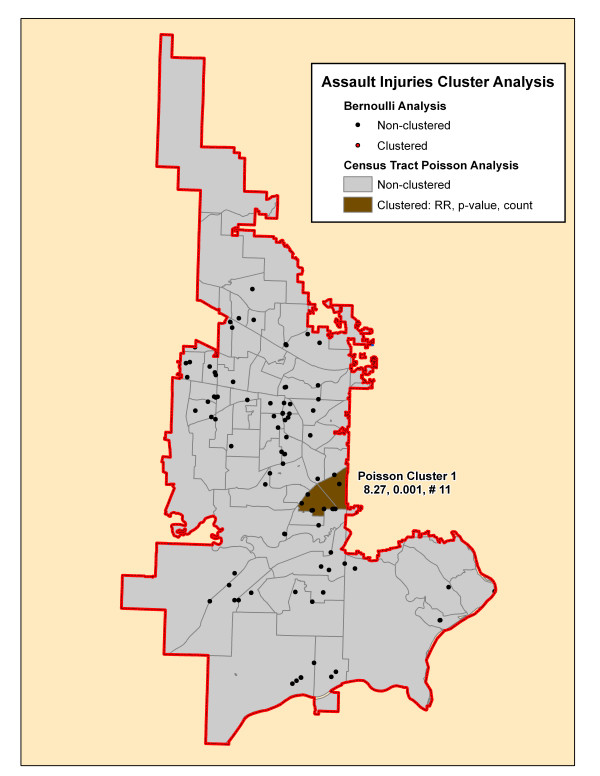
**Assault injuries cluster analysis**. The significant cluster by the SaTScan Poisson (brown-shaded census tract) is presented on this map. The cluster is identified by its rank followed by its relative risk (RR), associated p-value and the number of cases in the cluster.

Table 2 summarizes the results of the Bernoulli cluster analysis using SaTScan with the relative risk (RR), associated p-value, and number of cases included for each cluster. The table demonstrates the number of clusters and number of cases included in these significant clusters varied from a total of 13 clusters for motor vehicle collisions that included 58% of all cases to only one small cluster each for pedestrian and bicycle injuries and fall injuries. There were no significant clusters found for environmental and assault injury cohorts. Figures 4, 5, 6, 7, 8, 9 demonstrate the location of the Bernoulli clusters for each mechanism respectively and are discussed in more detail in the Results section "Comparison of Poisson and Bernoulli cluster analyses" below.

**Table 2:** Clusters identified by Bernoulli method stratified by mechanism

	**Relative Risk**	**P-value**	**Number in Cluster**
**Total Injuries (# 1997)**			
1^st^	2.16	0.005	25
2^nd^	2.17	0.008	24
3^rd^	1.41	0.005	154
4^th^	2.37	0.013	17
Total			220 (11%)
**Motor Vehicle Collision Injuries (# 413)**			
1^nd^	6.88	0.001	15
2^rd^	8.48	0.001	11
3^th^	3.20	0.001	33
4^th^	2.29	0.001	84
5^th^	10.44	0.005	6
6^th^	5.92	0.006	12
7^th^	6.61	0.006	10
8^th^	8.41	0.003	8
9^th^	11.80	0.016	5
10^th^	4.44	0.014	15
11^th^	2.98	0.013	27
12^th^	10.48	0.025	6
13^th^	6.56	0.021	9
Total			241 (58%)
**Pedestrian & Bicycle Injuries (# 219)**			
1^st^	14.11	0.005	5
2^nd^	3.71	0.013	18
Total			23 (10%)
**Fall Injuries (# 1035)**			
1^st^	3.38	0.001	18
Total			18 (2%)
**Environmental Injuries (# 236)**			
None			
**Assault Injuries (# 94)**			
None			

### Poisson cluster analysis

Table 3 shows the number of significant clusters obtained for all mechanisms combined and each mechanism separately denoting each cluster's relative risk, p-value, and number of cases included in the cluster. All had at least one significant cluster found with total injuries and motor vehicle collisions having a large part of cases included in clusters whereas the others had much fewer cases accounted for in clusters. Figures 4, 5, 6, 7, 8, 9 demonstrate the locations of the Poisson and Bernoulli clusters for each mechanism respectively and will be discussed in more detail under Results section "Comparison of Poisson and Bernoulli cluster analyses" below.

**Table 3:** Clusters identified by the Poisson method stratified by mechanism

	**Relative Risk**	**P-value**	**Number in Cluster**
**Total Injuries (# 1997)**			
1^st^	1.67	0.001	428
2^nd^	6.84	0.001	37
3^rd^	2.41	0.001	125
4^th^	2.36	0.001	57
Total			647 (32%)
**Motor Vehicle Collision Injuries (# 413)**			
1^nd^	2.80	0.001	130
2^rd^	2.25	0.024	30
3^th^	5.67	0.025	8
Total			168 (41%)
**Pedestrian & Bicycle Injuries (# 219)**			
1^st^	4.44	0.001	24
2^nd^	10.65	0.001	11
Total			35 (16%)
**Fall Injuries (# 1035)**			
1^st^	8.97	0.001	25
2^nd^	2.74	0.001	73
3^rd^	2.32	0.005	40
4rd	2.27	0.013	35
Total			173 (17%)
**Environmental Injuries (# 236)**			
1^st^	7.99	0.002	9
Total			9 (4%)
**Assault Injuries (# 94)**			
1^st^	8.27	0.001	11
Total			11 (12%)

### Comparison of Poisson and Bernoulli cluster analyses

Figures 4, 5, 6, 7, 8, 9 show the location of the significant Poisson and Bernoulli clusters for the total injuries cohort and each of the individual mechanisms respectively. Each cluster is labelled with its RR, p-value of that cluster RR and the number of cases contained in the cluster. In general, there is some geographical overlap of clusters by each method but there are some marked differences in the location of clusters identified. The comparison of each method on the same map highlights the strengths and weaknesses of each method and if the clusters overlap strengthens the impression that the area is a "hotspot." The two analyzes have different hypotheses since the Poisson method compares the injury rates to the underlying population using census tract data and the Bernoulli compares the location of events to a control group which may or may not be appropriate.

Figure 4 demonstrates the location of clusters for the total injuries cohort. There are an equal number of clusters found in each method but the Poisson method includes 32% of all cases in its clusters while the Bernoulli method only includes 11% of cases. Three of the Bernoulli clusters mostly overlap with one large Poisson cluster in the most urban part of the fire district while one Poisson cluster consisting of one large census tract has no proximate Bernoulli cluster.

There is even a starker difference in the respective cluster arrangement for the motor vehicle collision injury cohort with 13 clusters found in the Bernoulli method while only 3 found in the Poisson method in Figure 5. Again, there is some overlap in the core urban area and also some in the middle, western border but otherwise little overlap. The Bernoulli method has much smaller cluster sizes demonstrating perhaps a higher sensitivity in finding clusters since it uses precise incident locations.

Figure 6 maps the clusters for pedestrian and bicycle injuries with each method having two significant clusters that in this case do overlap. The number of cases included in the clusters is also similar and the RRs for the central clusters are the highest for its respective method.

The falls injuries cluster analysis (Figure 7) found only one cluster with the Bernoulli method and four with the Poisson method, one of which overlaps on the eastern boundary. The Poisson analysis resulted in having only one tract per cluster and overall contained 17% of cases while the small Bernoulli cluster had only 2% of the total.

Finally, Figures 8 and 9 show a single Poisson cluster containing one census tract for the environmental and assault injury cohorts, respectively. Each contains a small proportion of the total cases in the cohort. There are no significant Bernoulli clusters found for these mechanisms.

## Discussion

This study of pediatric injuries in a fire district database showed significant clustering for overall injuries and for each mechanism cohort for either the Poisson or Bernoulli method or both. Except for motor vehicle collisions, the majority of injuries occurred outside any identifiable clusters. The RR and corresponding p-value for most of the clusters are very significant so there is little doubt that these are high-risk areas. On the other hand, targeting injury prevention strategies only to these high risk areas may only have a minimal impact on the overall injury rates.

This has implications for the field of pediatric injury prevention. Injury prevention programs either free-standing or as part of a larger organization such as a fire department struggle with how to implement programs effectively. They have to decide which mechanisms to focus on, what age groups to include, and what geographic areas are highest risk. They also need to contend with whether there are effective preventative strategies available, [22,23] how much they cost, and are there any appropriate personnel or infrastructure to implement them. Most injury prevention programs have to compromise among these many factors. With a good GIS analysis of injury patterns, though, the programs should be able to make better decisions.

The clusters identified may be the first place to start some injury prevention activities since they have been identified as high-risk areas. These may be good places to establish pilot projects since stakeholders there may be more motivated to work on prevention activities and since the rates are already high it may be easier to show an effect for interventions. Since most injuries occur outside these clusters, the programs developed by these pilot projects need to be distributed throughout the organization's catchment area to have any appreciable effect on injury incidence. For example, the fire district could first focus on the two clusters of pedestrian and bicycle injuries and do further analysis of what age groups of children are most involved and what activities these children may be involved in leading to an increased rate of injury. A simple question to ask is whether the clusters seem to be proximate to schools, parks or commercial areas. One study found increased pedestrian injuries in proximity to a school in four Californian communities.[24] Once the location of clusters of pedestrian injuries are found there are proven interventions that can be done at schools to decrease the incidence.[25] The present study demonstrates only two significant clusters of pedestrian injuries in the study area consistent with a previous study in Montréal.[6]

The major limitation of this study is that the original data collection was for patient care and not an injury prevention analysis. Trying to obtain similar data through a specific injury prevention research project would be very expensive and take several years to complete. This study used data fields that should be accurate since they are also needed for documentation of patient care. Most similar analyses come from secondary data sources due to resource constraints. As emergency medical service agencies engage in more injury prevention strategies they will collect more appropriate data points to manage these effectively, allowing better research. This data also excluded any injuries that did not generate a 9-1-1 call but may have been seen in a primary care office or emergency department. The relative distribution of where these children are seen first varies with the severity of injury, where the injury occurs, and what mechanism is involved.[5,21,23] This dataset should account for most of the serious non-intentional injuries in this population. Except for a few select geographical areas in the United States, a comprehensive injury data collection process is not in place and one has to depend on secondary and limited sources.

Each method of cluster analysis in SaTScan has potential strengths and weaknesses. Certainly the geographical overlap of both methods was less than perfect with each finding different number of significant clusters for most mechanisms. The Poisson method had to rely on census tract level population data that outside the core urban areas have less regular shapes and population densities. In addition, since the fire district's boundary did not always correspond to census tract boundaries multiple portions of census tract polygons were used for analysis leading to possible distortion due to uneven population distributions. SaTScan uses a circular window on census tract centroids to determine potential cluster boundaries which may not represent the population at risk in a realistic fashion. The injury rates are relatively low so attempting aggregations at a smaller population size such as census blocks may lead to very low rates for analysis.

Using the Bernoulli method in SaTScan with the controls being medical cases can be criticized. The two cohorts did differ in some demographic factors that may influence the cluster analysis results. The strength of this approach is using cases and controls drawn from a sample of the population at-risk that use the 9-1-1 emergency response system. One would be more certain of overlapping clusters identified by both methods to be real and one might concentrate on these for further analysis. Even if one could more accurately map the population distribution by using dysametric methods and remote sensing, for example, this will still not take into account the population at-risk that travels through, goes to school or day care in or works in the study area. Estimating the distribution of this population would be a huge undertaking with a limit in available data and statistical methods to analyze it.

One could argue that for most injuries especially ones occurring on the road system it would be more appropriate to use network analysis to find clusters. Currently, cluster analysis on a network using a stochastic model is in its early stages.[26,27] This would be a logical next step in the analysis of injury data especially road-related ones.

## Conclusion

In this study of pediatric injuries involving a fire district 9-1-1 response, there were identifiable high-risk clusters found for all injury mechanisms combined and each mechanism separately by at least one method of cluster detection. There are strengths and weaknesses to each method of cluster detection. Finding these clusters is the first step in targeting injury prevention interventions to decrease the incidence. More detailed GIS and demographic analysis will further refine possible strategies and allow more rational choices. Other methods of analysis should be attempted on the location of incidents involving injuries.

## Abbreviations

RR: relative risk; TVF&R: Tualatin Valley Fire and Rescue.

## Competing interests

The author declares that they have no competing interests.

## Authors' contributions

The author is responsible for all the data analysis and writing of the manuscript.
